# Cullin 4A/protein arginine methyltransferase 5 (CUL4A/PRMT5) promotes cell malignant phenotypes and tumor growth in nasopharyngeal carcinoma

**DOI:** 10.1080/21655979.2022.2054756

**Published:** 2022-03-25

**Authors:** Xiuying Sun, Jinhui Zhou, Zhicun Zhang

**Affiliations:** Department of Otolaryngology, The Affiliated Huai’an No. 1 People’s Hospital of Nanjing Medical University, Huai’an, Jiangsu, China

**Keywords:** Cullin 4A, protein arginine methyltransferase 5, p65, nasopharyngeal carcinoma, C666-1 cell

## Abstract

Targeted therapy is an important therapeutic strategy currently, however, the development of targeted therapy for nasopharyngeal carcinoma (NPC) is relatively lagging. Cullin 4A (CUL4A) was reported to be overexpressed in NPC; nevertheless, the specific role of CUL4A remains unrevealed. NPC cells and tumor-bearing mice were cultivated to explore the role and mechanism of CUL4A in NPC. After evaluating CUL4A levels in NPC cells, functional experiments were carried out to investigate the effects of CUL4A knockdown and overexpression on cell proliferative, invasive and migratory aptitude as well as NF-κB signaling. Following the GeneMANIA database predicted that protein arginine methyltransferase 5 (PRMT5) was downstream of CUL4A, the mediated role of PRMT5 in the regulation of CUL4A on cells was then determined. Moreover, the tumor volumes and weights of tumor-bearing mice were recorded, and the levels of proliferation-, migration-, and NF-κB signaling-related proteins in the tumor were determined. Herein, CUL4A was enhanced in NPC cells, and its knockdown and overexpression separately suppressed and promoted cell proliferative, invasive, and migratory aptitude as well as NF-κB signal activation. Novelty, PRMT5 knockdown reversed the influences of CUL4A overexpression on these aspects. In addition, its knockdown likewise reversed the facilitating impact of CUL4A expression on tumor growth and declined the expression levels of proliferation-, migration-, and NF-κB signaling-related protein in the tumor. Together, this paper indicated that CUL4A promoted the proliferative, invasive, and migratory aptitude of NPC cells as well as tumor growth by promoting PRMT5 to activate NF-κB signaling.

## Graphical Abstract

**Figure:**
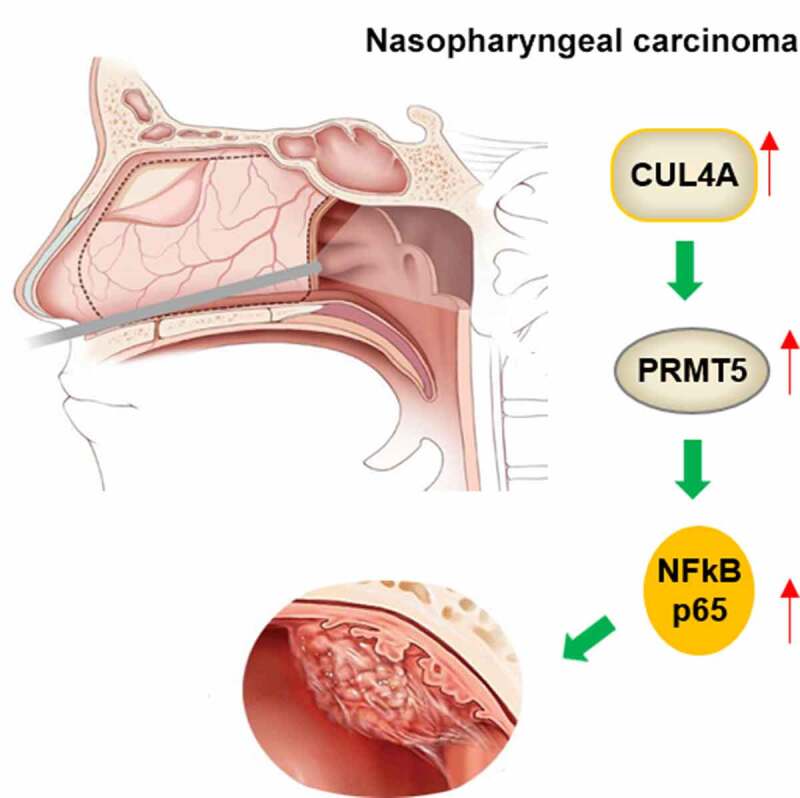

## Introduction

Nasopharyngeal carcinoma (NPC) is an epithelial malignant tumor that occurs in the nasopharynx. It originates from the pharyngeal recesses and easily invades adjacent tissues and lymph nodes [1]. The degree of malignancy is relatively high and distant metastasis can occur [2]. NPC was divided into keratinizing, non-keratinizing, and basaloid squamous cell carcinoma by World Health Organization according to its histology [3]. Numerous epidemiological surveys indicate that Epstein-Barr virus (EBV) infection is the most universal cause, meanwhile, environmental and genetic factors may also be involved [4]. EBV infection is common in the non-keratinizing type, taking up over 90% of cases; EBV is rare in keratinizing type with a relatively favorable prognosis [5]. Because NPC has an insidious onset and is prone to regional lymph nodes and distant hematogenous metastasis, the most patients are in the middle and late stages when the disease is clinically diagnosed [6]. Intensity-modulated radiotherapy (IMRT) can significantly improve the local control rate of NPC [7], however, distant metastasis, especially to the lung, bone, and liver, is still an important reason for failed treatment [2]. Molecular targeted therapy of tumors has become a hot spot in the field of tumor research, and it is also an important therapeutic strategy currently. Compared with other tumors, the development of targeted therapy of NPC is relatively lagging.

Being a bridging protein in the ubiquitin ligase complex, Cullin 4A (CUL4A) is involved in the ubiquitination degradation and ubiquitination modification of proteins *in vivo* and determines the specificity of substrate proteins [8]. Previous studies have revealed that CUL4A is involved in various physiological activities of cells, including the regulation of cell cycle, the transduction of signal, the repair of damaged DNA, the methylation of histone, the regulation of transcription as well as the activation of an oncogene [9]. Therefore, abnormal expression of CUL4A protein may affect the physiological activities of cells and lead to tumorigenesis [10]. Overexpression of CUL4A protein was initially found in primary breast cancer, suggesting that CUL4A overexpression has closed relation to the development of breast cancer [11]. Subsequent studies found that CUL4A overexpression also occurred in the prostate, colon, liver, as well as lung cancer [^12–15^]. Of note, it was evidenced that CUL4A was overexpressed in NPC [16], nonetheless, the role of CUL4A, as well as its mechanism, has not been revealed.

Therefore, this study selected NPC cells and constructed tumor-bearing mouse model to explore the role and mechanism of CUL4A in NPC. Exploring molecular therapeutic targets is of great significance for enriching the comprehensive treatment strategies for patients with NPC and improving the therapeutic efficacy.

## Materials and methods

### Cell culture

Human nasopharyngeal epithelial NP69 cells (Biobw, Beijing, China) and NPC cells C666-1, 5–8 F, 6–10B (Meisen, Hangzhou, China), and SUNE-1 (EK-Bioscience, Shanghai, China) were cultured in RPMI-1640 (Gibco; Thermo Fisher Scientific) supplemented with 10% FBS (Gibco) and 1% P/S in an incubator set at 37°C and 5% CO_2_ [17].

### Cell transfection

CUL4A knockdown was achieved by transfection with small interfering (si)RNA targeting CUL4A (siRNA-CUL4A-1/2), and a non-targeted siRNA was used as negative control (siRNA-NC). CUL4A overexpression was achieved through the transfection with pcDNA3.1 vector containing CUL4A (Ov-CUL4A), and the empty vector was used as Ov-NC. PRMT5 knockdown was achieved by transfection with short hairpin RNA (shRNA) targeting PRMT5 (shRNA-PRMT5-1/2), and a scrambled shRNA was used as shRNA-NC. All these plasmids were provided by GenePharma (Shanghai, China). The transfection [18] was carried out with the aid of Lipofectamine® 2000 (Invitrogen; Thermo Fisher Scientific).

### Preparation of tumor transplants

All procedures followed the ethical guidelines of The Affiliated Huai’an No. 1 People’s Hospital of Nanjing Medical University and were approved by The Research and Education Animal Use and Care Committee. Great efforts have been made to relieve animal suffering. The experiments were carried out using 18 adult male Balb/c nude mice [19] (age, 4–6 weeks; weight, 16–18 g; Cyagen Biosciences, Jiangsu, China) which were housed at a constant ambient temperature of 21 ± 2°C and relative humidity of 55 ± 5%, with a 12-h light/dark cycle, and free access to food and water. For each group (Ov-NC, Ov-CUL4A, and Ov-CUL4A + shRNA-PRMT5), the resuspension of cells (2 × 10^6^) was separately conducted in a 0.2-ml volume and the cell suspension was injected using a syringe. The weights of the mice along with the size of the tumors were recorded every 3 days from the fourth day. A total of 28 days later, after the nude mice were euthanized using an overdose of sodium pentobarbital (100 mg/kg, ip) followed by cervical dislocation, the tumor tissue was taken out for the subsequent assays.

### Western blotting

Extracted proteins from tumor tissue or cells by RIPA lysis buffer (Solarbio, Beijing, China) were quantified with the adoption of a BCA assay (Solarbio). After the separation with 10% polyacrylamide gel, the transfer of proteins (25 μg) was carried out into PVDF membranes (Millipore). Prior to the incubation with primary antibodies at 4°C, the blocking of membranes with skimmed milk was operated. Thereafter, membranes were cut in strips, HRP-conjugated secondary antibody was applied to incubate the strips. Taking advantage of an ECL detection reagent (Millipore), the blots were tracked and ImageJ version 1.52 software was utilized to analyze the obtained data [20]. The details of the antibodies are presented in Table 1.

**Table 1.** Antibodies used for western blotting

Antibody	Catalog number	Host	Dilution ratio	Company
CUL4A	GTX33129	Rabbit	1:1,000	GeneTex
PRMT5	GTX116004	Rabbit	1:1,000	GeneTex
MMP12	ab52897	Rabbit	1:5,000	Abcam
MMP9	ab283575	Rabbit	1:1,000	Abcam
Ki67	ab16667	Rabbit	1:1,000	Abcam
PCNA	ab29	Rabbit	1:1,000	Abcam
Phospho-p65	AF5875	Rabbit	1:1,000	Beyotime
p65	AF0246	Rabbit	1:1,000	Beyotime
IKBα	ab97783	Rabbit	1:5,000	Abcam
Histone3	Gtx122148	Rabbit	1:5,000	GeneTex
HRP anti-rabbit lgG	A0208	goat	1:1,000	Beyotime

### Reverse transcription-quantitative PCR (RT-qPCR)

The synthesization of RNA isolated by TRIzol® reagent (Invitrogen) into cDNA was operated with PrimeScript™ RT Master Mix (Takara, Beijing, China). qPCR was carried out with the application of the QuantiTect SYBR-Green PCR kit (Qiagen). The data were quantified with the ∆∆Cq method [21] following normalization against GAPDH. The following were the applied primers: CUL4A forward, 5’-AGGCACAGATCCTTCCGTTT-3’ and reverse, 5’-TCCTGCCAGCACGTGTTAAT-3’; PRMT5 forward, 5’-CTGTCTTCCATCCGCGTTTCA-3’ and reverse, 5’-GCAGTAGGTCTGATCGTGTCTG-3’; and GAPDH forward, 5’- GACTCATGACCACAGTCCATGC-3’ and reverse, 5’-AGAGGCAGGGATGATGTTCTG-3’.

### Cell Counting Kit-8 (CCK-8) assay

Cells (5 × 10^3^ cells/well) that plated into a 96-well plate were cultured for 24, 48, and 72 h. For each time point, the CCK-8 solution (Dojindo Molecular Technologies) was added to each well [22]. Absorbance was then tracked under the application of a microplate reader (λ = 450 nm, Bio-Rad Laboratories).

### Co-Immunoprecipitation (Co-IP)

The lysis of cells was operated on ice for 10 min, following which was the centrifugation at 13,000 x g for 10 min and the collection of supernatants. Subsequently, the lysate (500 µg/ IP) was supplemented with the 2.5 µg CUL4A or PRMT5 antibody and 10 µl protein A + G magnetic beads (Beyotime, Shanghai, China) followed by gentle rotation at room temperature for 2 h. The supernatant was removed by magnetic force and the magnetic beads with the added 1X SDS sample buffer were boiled for 5 min for western blotting [23].

### Wound healing assay

Cells (5 × 10^5^ cells/well) in six-well plates were cultured until ~90% confluence. A 200-μl sterile pipette tip was applied to create a wound in the cell monolayer. Following the rinse with PBS, the cells were starved for 24 h [24]. Under an inverted microscope (x100; Olympus Corporation), the images were visualized at 0 and 24 h.

### Transwell assay

Cells (3 × 10^4^ cells) were suspended in 1 ml serum-free medium. The upper chamber was pre-coated with Matrigel (Sigma-Aldrich) and cells were then cultivated in the upper chamber with 0.1 ml cell suspension in each well. RPMI-1640 which contains 20% FBS was applied to perfuse the lower chamber. Following 24 h of incubation, the fixation and staining with 4% paraformaldehyde (Biobw) and 0.5% crystal violet solution (Yeasen, Shanghai, China) were operated [25]. Finally, the stained cells were totaled under an inverted microscope (x100).

### Colony formation assay

Cells were inoculated into culture dishes at a density of 500 cells/dish. After discarding the supernatants, the cells were fixed with 4% paraformaldehyde (Biobw). The fixative solution was then replaced with crystal violet (Yeasen) to stain the cells. A cluster of >50 cells was considered a colony [26].

### Bioinformatics and statistical analysis

GeneMANIA (http://genemania.org/) online databases were used to search for the association between known proteins [27]. Data that demonstrated as the mean ± SD were analyzed with Prism 8.0 software (GraphPad Software). To show differences among multiple groups, one-way ANOVA followed by Tukey’s post hoc test was employed. P less than 0.05 is judged to be of statistical significance.

## Results

This study used NPC cells and tumor-bearing mouse models to explore the role and mechanism of CUL4A in NPC. CUL4A expression was enhanced in NPC cells, and its knockdown and overexpression separately suppressed and promoted cell proliferative, invasive, and migratory aptitude as well as NF-κB signal activation. Novelty, PRMT5 knockdown reversed the influences of CUL4A overexpression on these aspects. Moreover, its knockdown also reversed the facilitating impact of CUL4A expression on tumor growth and declined the expression levels of proliferation-, migration-, and NF-κB signaling-related protein in the tumor. Together, these results indicated that CUL4A promoted the proliferative, invasive, and migratory aptitude of NPC cells as well as tumor growth by promoting PRMT5 to activate NF-κB signaling.

## CUL4A expression level impacts NPC cell proliferation

**Figure 1.**
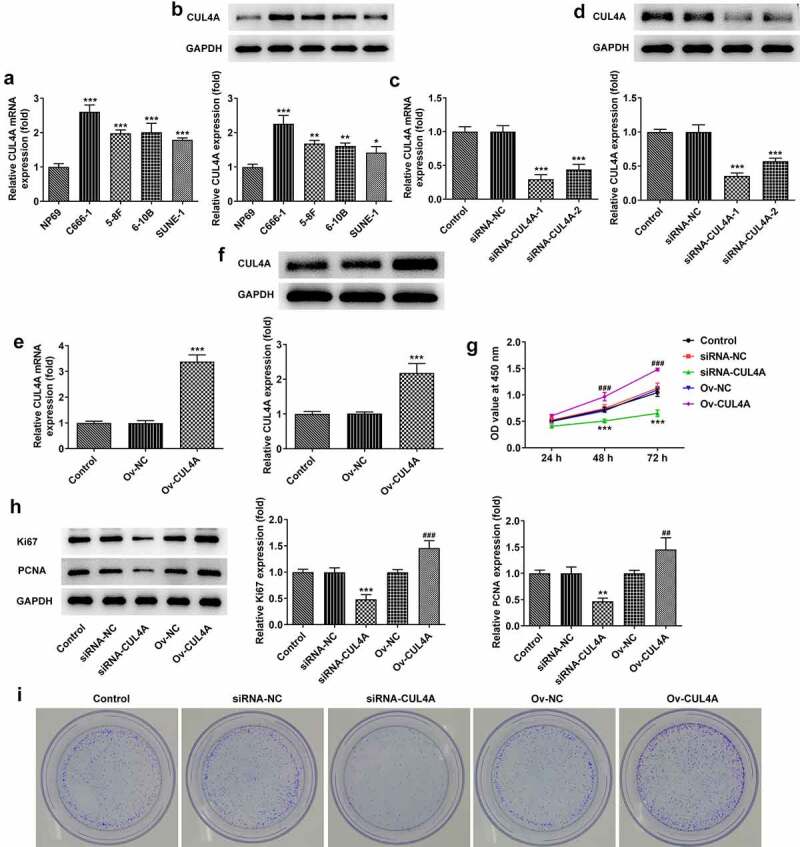
**CUL4A expression level impacts NPC cell proliferation** (a) The expression levels of CUL4A in NP69, C666-1, 5–8 F, 6–10B, and SUNE-1 cells were determined using RT-qPCR and (b) western blotting. *P < 0.05, **P < 0.01, ***P < 0.001 vs. NP69. (c) The efficacy of CUL4A knockdown was assessed using RT-qPCR and (d) western blotting. ***P < 0.001 vs. siRNA-NC. (e) The efficacy of CUL4A overexpression was assessed using RT-qPCR and (f) western blotting. ***P < 0.001 vs. Ov-NC. (g) Cell proliferation in each group was determined using the CCK-8 assay. (h) The expression level of Ki67 and PCNA was determined using western blotting. (i) Colony-forming efficiency was assessed by colony formation assay. **P < 0.01, ***P < 0.001 vs. siRNA-NC; ^##^P < 0.01, ^###^P < 0.001 vs. Ov-NC.

The levels of CUL4A in NP69, C666-1, 5–8 F, 6–10B, and SUNE-1 cells were assessed by RT-qPCR along with western blotting (Figure 1a-b). Higher expression levels were found in NPC cells compared with NP69 cells. To highlight the probable effects of CUL4A level on NPC cells, we adopted C666-1 cells for ensuing experiments. The C666-1 cells were transfected with siRNAs or plasmids to knock down or elevate the expression of CUL4A, respectively. The transfection efficacy was assessed using RT-qPCR and western blotting (Figure 1c-f). The level of CUL4A was effectively down-regulated in the siRNA-CUL4A-1/2 groups, and the siRNA-CUL4A-1 group was selected for the following assays due to the relatively lower level. In addition, CUL4A was effectively overexpressed in the Ov-CUL4A group. Cell proliferation in the knockdown and overexpression groups was determined using the CCK-8 assay (Figure 1g). During the period of 24, 48, 96 h, the proliferative ability of cells in the siRNA-CUL4A group slowed down, whereas the proliferation in the Ov-CUL4A group accelerated. Moreover, the levels of Ki67 and PCNA were evaluated by western blotting (Figure 1h). Their levels were declined in the siRNA-CUL4A group and elevated in the Ov-CUL4A group. Colony-forming efficiency was likewise assessed by plate colony formation assay (Figure 1i). The colony-forming efficiency in the siRNA-CUL4A group was obviously decreased, while that in the Ov-CUL4A group was improved.

## CUL4A expression level impacts NPC cell migration and invasion, and NF-κB signaling

**Figure 2.**
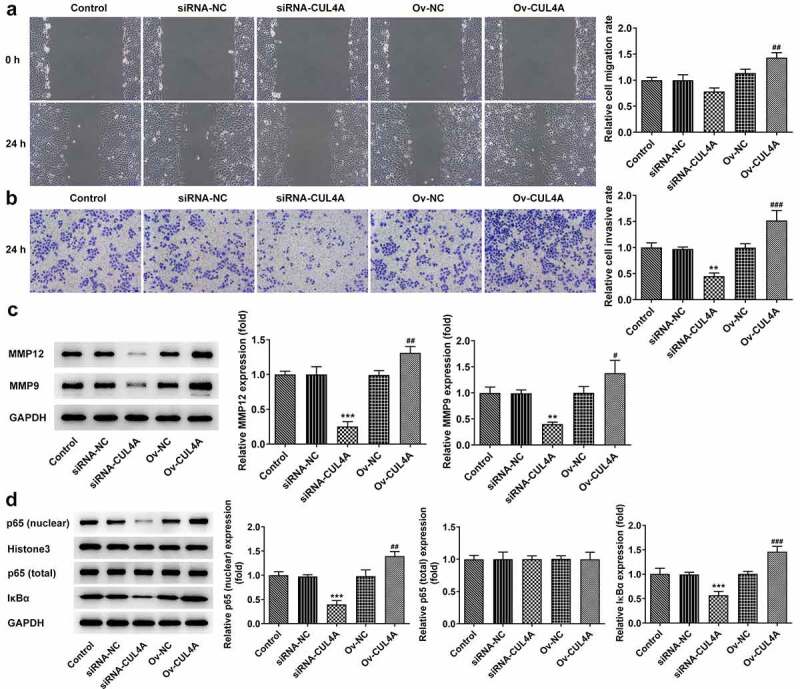
**CUL4A expression level impacts NPC cell migration and invasion, and NF-κB signaling** (a) Cell migration was determined using wound healing assay. (b) Cell invasion was determined using Transwell assay. (c) The expression levels of MMP12 and MMP9 were determined using western blotting. (d) The expression level of nuclear p65, total p65, and IκBα were determined using western blotting. **P < 0.01, ***P < 0.001 vs. siRNA-NC; ^#^P < 0.05, ^##^P < 0.01, ^###^P < 0.001 vs. Ov-NC.

The cell migratory and invasive aptitude were assessed employing wound healing along with Transwell assay (Figure 2a-b). The migration rate of cells in the siRNA-CUL4A group was slightly decreased and significantly increased in the Ov-CUL4A group. The invasion rate of cells was significantly decreased in the siRNA-CUL4A group and increased in the Ov-CUL4A group, respectively. In addition, with the adoption of western blotting, the MMP12 and MMP9 levels were detected (Figure 2c). Their levels were markedly declined in the siRNA-CUL4A group and increased in the Ov-CUL4A group. Furthermore, as Figure 2d depicted, the levels of nuclear p65 and IκBα were conspicuously descended in the siRNA-CUL4A group and elevated in the Ov-CUL4A group.

## CUL4A regulates PRMT5 to impact NF-κB signaling

**Figure 3.**
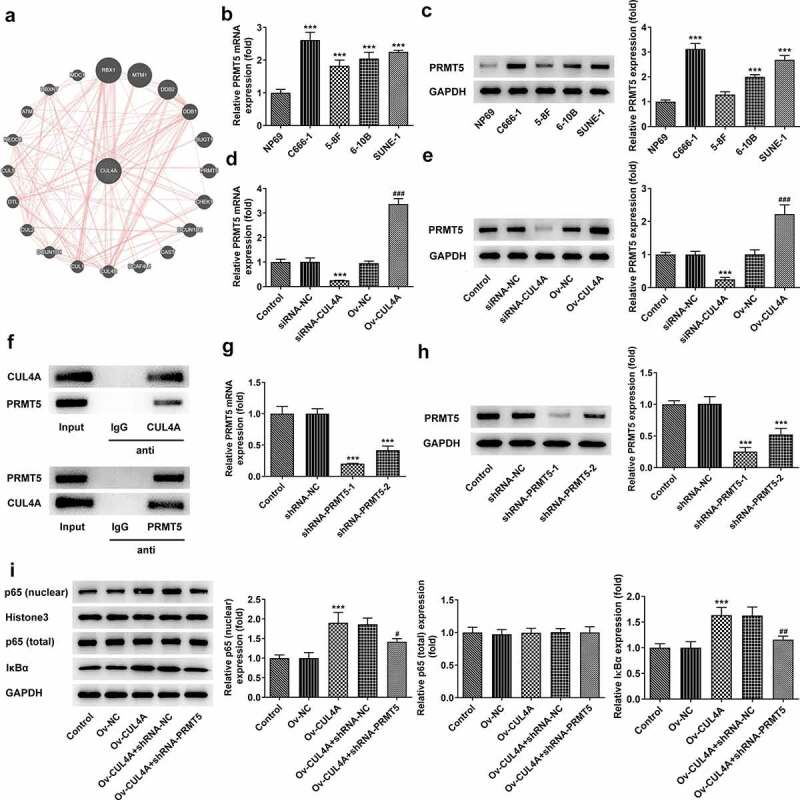
**CUL4A regulates PRMT5 to impact NF-κB signaling** (a) The GeneMANIA database was applied to search proteins associated with CUL4A. (b) The expression level of PRMT5 in NP69, C666-1, 5–8 F, 6–10B, and SUNE-1 cells were determined using RT-qPCR and (c) western blotting. ***P < 0.001 vs. NP69. (d) The expression level of PRMT5 in the transfected cells was determined using RT-qPCR and (e) western blotting. ***P < 0.001 vs. siRNA-NC; ^###^P < 0.001 vs. Ov-NC. (f) The association between CUL4A and PRMT5 was verified using the Co-IP assay. (g) The efficacy of PRMT5 knockdown was confirmed with RT-qPCR and (h) western blotting. ***P < 0.001 vs. shRNA-NC. (i) The expression level of nuclear p65, total p65, and IκBα were determined using western blotting. ***P < 0.001 vs. Ov-NC; ^#^P < 0.05, ^##^P < 0.01 vs. Ov-CUL4A + shRNA-NC.

To further figure out the mechanism of CUL4A, the GeneMANIA database was applied to search proteins associated with CUL4A (Figure 3a). PRMT5 was found to be co-expressed with CUL4A. Hence, the levels of PRMT5 in NP69, C666-1, 5–8 F, 6–10B, and SUNE-1 cells were assessed with RT-qPCR and western blotting (Figure 3b-c). The level of PRMT5 was enhanced in the NPC cells. Moreover, the PRMT5 level was markedly declined in the siRNA-CUL4A group and elevated in the Ov-CUL4A group (Figure 3d-e). Afterward, the association between CUL4A and PRMT5 was verified using the Co-IP assay (figure 3f). The results indicated that CUL4A and PRMT5 could bind to each other and co-precipitate. Following confirming the efficacy of shRNA-PRMT5 with RT-qPCR along with western blotting (Figure 3g-h), shRNA-PRMT5-1 and Ov-CUL4A were co-transfected into C666-1 cells. The levels of nuclear p65, total p65, and IκBα were evaluated with the application of western blotting (Figure 3i). PRMT5 knockdown reversed the elevated expression levels of nuclear p65 and IκBα caused by CUL4A overexpression.

## CUL4A regulates PRMT5 to impact NPC cell malignant phenotypes

**Figure 4.**
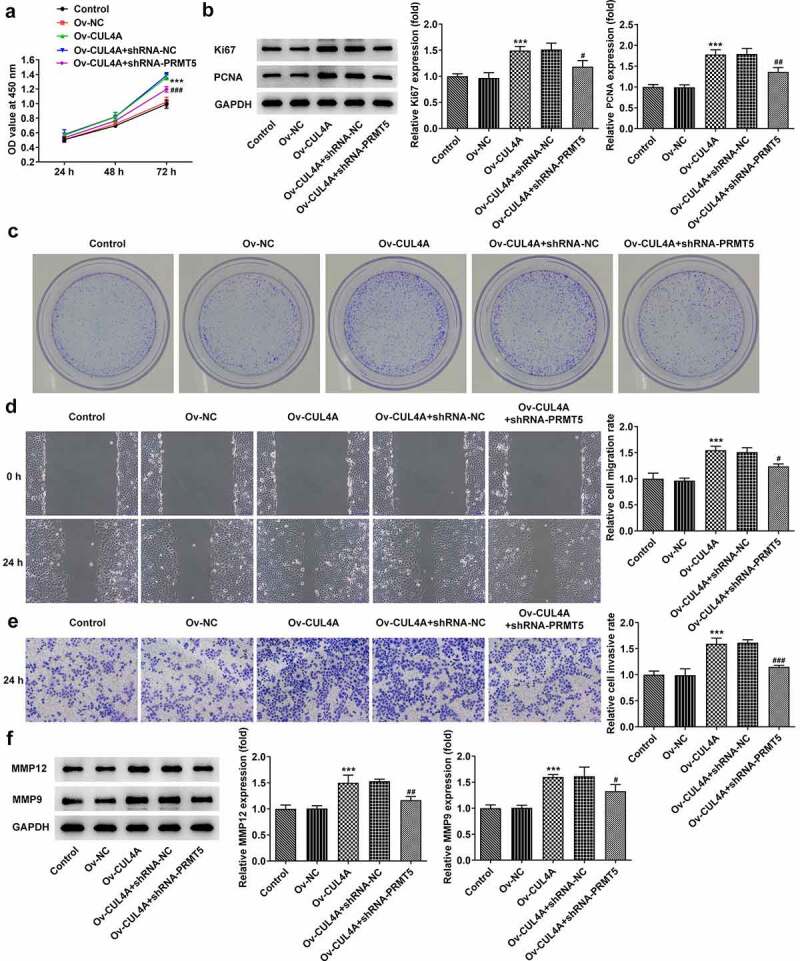
**CUL4A regulates PRMT5 to impact NPC cell malignant phenotypes** (a) Cell proliferation level was determined with the CCK-8 assay. (b) The expression level of Ki67 and PCNA was determined using western blotting. (c) Colony-forming efficiency was assessed by colony formation assay. (d) Cell migration was determined using a wound healing assay. (e) Cell invasion was determined using Transwell assay. (f) The expression levels of MMP12 and MMP9 were determined using western blotting. ***P < 0.001 vs. Ov-NC; ^#^P < 0.05, ^##^P < 0.01, ^###^P < 0.001 vs. Ov-CUL4A + shRNA-NC.

The influences of PRMT5 knockdown on the regulatory roles of CUL4A in cell malignant phenotypes were evaluated. Cell proliferative ability was firstly tracked by CCK-8 assay (Figure 4a), western blotting (Figure 4b), as well as colony formation assay (Figure 4c). PRMT5 knockdown reversed the promotion of cell proliferation resulting from CUL4A overexpression and simultaneously reduced the expression levels of Ki67 and PCNA. Moreover, the colony-forming efficiency in the Ov-CUL4A + shRNA-PRMT5 group was declined compared with the Ov-CUL4A + shRNA-NC group. Next, the capacity of cells to migrate and invade was determined using wound healing (Figure 4d), Transwell assay (Figure 4e), and western blotting (figure 4f). PRMT5 knockdown likewise reversed the promoted migration and invasion caused by CUL4A overexpression, meanwhile, it reduced the expression levels of MMP12 and MMP9.

## CUL4A regulates PRMT5 to promote tumor growth

**Figure 5.**
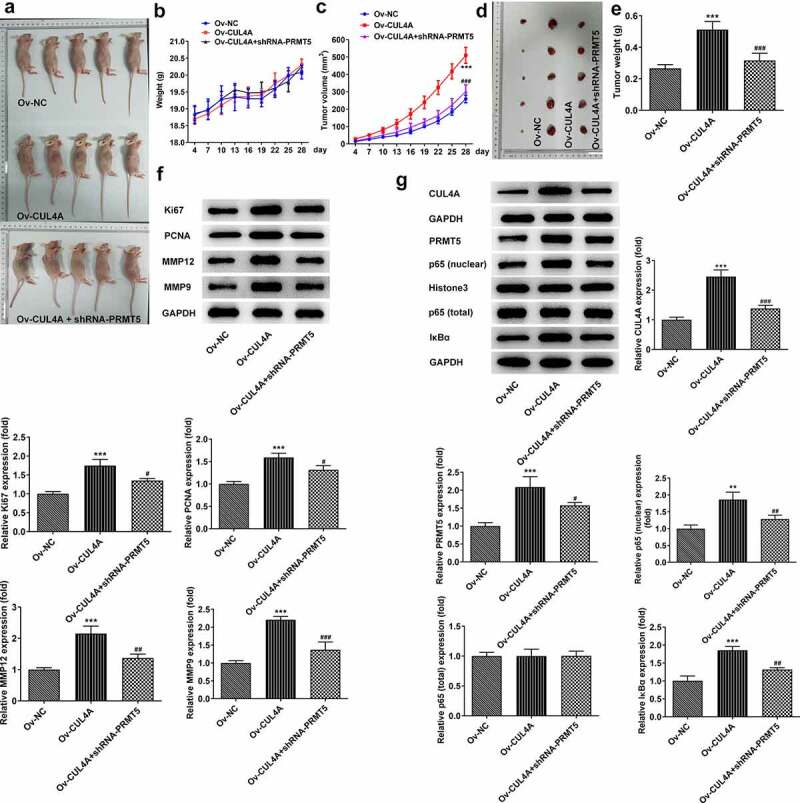
**CUL4A regulates PRMT5 to promote tumor growth** (a) Photo of tumor-bearing mouse models. (b) The body weights of mice and (c) the volumes of tumors were recorded over 28 days. (d) Photo of the dissected tumor. (e) The weights of dissected tumors were recorded. (f) The expression levels of Ki67, PCNA, MMP12, and MMP9 in the tumor were determined using western blotting. (g) The expression level of CUL4A, PRMT5, nuclear p65, total p65, and IκBα in the tumor was determined using western blotting. **P < 0.01, ***P < 0.001 vs. Ov-NC; ^#^P < 0.05, ^##^P < 0.01, ^###^P < 0.001 vs. Ov-CUL4A.

Based on cellular experiments, we performed *in vivo* study using tumor-bearing mouse models. The body weights of mice (Figure 5a-b) and the volumes of tumors (Figure 5c-d) were recorded over 28 days. Overall, the body weights presented fluctuating upward trends. Compared with the Ov-NC group, the weight of the mice in the Ov-CUL4A group was lower for the first 13 days and surpassed in the following days. Compared with the Ov-CUL4A group, the weight of mice in the Ov-CUL4A + shRNA-PRMT5 group was higher in the first 20 days, and lower in the remaining days. The tumor volume in the Ov-CUL4A group increased sharply and was significantly larger than that in the Ov-NC group on the day of stripping. The tumor volume in the Ov-CUL4A + shRNA-PRMT5 group increased slowly and was tremendously smaller than that in the Ov-CUL4A group on the day of stripping. The tumor weight in the Ov-CUL4A group was also greatly larger than that in the Ov-NC group, and PRMT5 knockdown greatly inhibited the growth of tumor weight (Figure 5e). The levels of Ki67, PCNA, MMP12, and MMP9 in the tumor were assessed by western blotting (figure 5f). Their levels in the tumor of the Ov-CUL4A group were elevated and partly reversed in the Ov-CUL4A + shRNA-PRMT5 group. Furthermore, the levels of CUL4A, PRMT5, nuclear p65, total p65, and IκBα in the tumor were assessed by western blotting (Figure 5g). CUL4A overexpression promoted intratumoral PRMT5 levels, as well as nuclear p65 and IκBα levels, whereas PRMT5 knockdown reduced their levels.

## Discussion

The incidence of NPC has unique geographical distribution characteristics, with the highest incidence in China and Southeast Asian countries, followed by North Africa, and the least in Europe, America, and Oceania [28]. This distribution is thought to be relevant to racial genetics, consumption of pickled foods, high levels of nickel in soil and water, and climate suitable for EBV survival. EBV is best suited to survive in hot and humid conditions [4,29]. As aforementioned, its infection is intimately associated with the occurrence and advancement of NPC. EBV DNA level has been identified as markers of tumor burden, recurrence monitoring, and prognosis in NPC [30]. The C666-1 cells, majorly studied in this paper, are EBV-positive cells [31], and the expression level of CUL4A is higher in them compared with other cells. As NPC is prone to metastasis, this study evaluated the effects of low and high CUL4A expression on C666-1 cell proliferative, invasive and migratory aptitude, respectively. The results indicated that down-regulated and up-regulated CUL4A expression separately inhibited and promoted cell proliferation, invasion, and migration. Although we found that CUL4A has a regulatory effect on cells as above-mentioned, the pathway mediated by it remains unknown, hence, we continued to investigate the downstream.

EBV can establish latent or lytic infection in host cells, both of which are involved in the carcinogenesis of NPC. Among them, EBV infection of NPC is dominantly based on latent infection [32]. EBV-encoded latent membrane protein 1 is the primary oncogenic protein of NPC, and many of its downstream events are mediated through activation of NF-κB [33]. Moreover, ubiquitination plays a very important role in the activation of the NF-κB signaling pathway and participates in the degradation of IκB protein [34]. Hence, we speculated that CUL4A could regulate NPC cells through the activation of the NF-κB signaling pathway. The results of this study display that CUL4A can promote the activation of NF-κB signaling in NPC cells, and inhibit its activity when its expression is knocked down. Activated NF-κB is an integral part of inducing tumor formation, and it has been suggested that NF-κB signaling is involved in the malignant behavior of NPC cells [35].

Afterward, we screened the GeneMANIA database and found that CUL4A can co-express with PRMT5. PRMT5 has been testified to be enhanced in NPC tissues and cells, and its knockdown can enhance the radiosensitivity [36]. We co-transfected CUL4A overexpression plasmid and PRMT5 interference plasmid into C666-1 cells and injected the cells subcutaneously into nude mice. We found that PRMT5 knockdown inhibited cell migration and invasion and slowed tumor growth in nude mice. PRMT5 is an emerging epigenetic enzyme that regulates the transcription of target genes through di-methylation of histone residues [37]. At present, there are highly selective PRMT5 inhibitors developed with PRMT5 as the target, which are used for the treatment of malignant solid tumors and hematological tumors, and have been approved for clinical trials [38]. This suggests that the use of PRMT5 as a target for the treatment of NPC is of great practical significance.

## Conclusion

Taken together, this paper indicates that CUL4A promotes the proliferation, migration, invasion of NPC cells, and tumor growth by promoting PRMT5 to activate NF-κB signaling. This finding revealed the mechanism of CUL4A/PRMT5 in NPC and laid a theoretical foundation for the selection of therapeutic targets. It is hoped that this research will advance the development of targeted drugs for the treatment of NPC.

## References

[1] Li H, Huang C, Chen Q, et al. Lymph-node Epstein-Barr virus concentration in diagnosing cervical lymph-node metastasis in nasopharyngeal carcinoma.Eur Arch Otorhinolaryngol. . 2020 Sep; 277 9: 2513–2520 32240363 10.1007/s00405-020-05937-5

[2] Sun XS, Li XY, Chen QY, et al. Future of Radiotherapy in Nasopharyngeal Carcinoma. Br J Radiol. 2019 Oct;92(1102):20190209. 31265322 10.1259/bjr.20190209 PMC6774595

[3] Holmes BJ, Wenig BM. Virus-associated carcinomas of the head & neck: update from the 2017 WHO classification. Ann Diagn Pathol. 2019 Feb;38:29–42. 30415111 10.1016/j.anndiagpath.2018.10.008

[4] Lee HM, Okuda KS, González FE, et al. Current Perspectives on Nasopharyngeal Carcinoma. Adv Exp Med Biol. 2019;1164:11–34. 31576537 10.1007/978-3-030-22254-3_2

[5] Guan S, Wei J, Huang L, et al. Chemotherapy and chemo-resistance in nasopharyngeal carcinoma. Eur J Med Chem. 2020 Dec 1;207:112758. 32858472 10.1016/j.ejmech.2020.112758

[6] Hong M, Tang K, Qian J, et al. Immunotherapy for EBV-Associated Nasopharyngeal Carcinoma. Crit Rev Oncog. 2018;23(3–4):219–234. 30311576 10.1615/CritRevOncog.2018027528

[7] Lee AWM, Ng WT, Chan JYW, et al. Management of locally recurrent nasopharyngeal carcinoma. Cancer Treat Rev. 2019 Sep;79:101890. 31470314 10.1016/j.ctrv.2019.101890

[8] Wang X, Chen T. CUL4A regulates endometrial cancer cell proliferation, invasion and migration by interacting with CSN6. Mol Med Rep. 2021 Jan;23(1):23. 33179082 10.3892/mmr.2020.11661 PMC7673334

[9] Ashok C, Owais S, Srijyothi L, et al. A feedback regulation of CREB activation through the CUL4A and ERK signaling. Med Oncol. 2019 Jan 21 36(2):20. 30666499 10.1007/s12032-018-1240-2

[10] Cui H, Wang Q, Lei Z, et al. DTL promotes cancer progression by PDCD4 ubiquitin-dependent degradation. J Exp Clin Cancer Res. 2019 Aug 13 38(1):350. 31409387 10.1186/s13046-019-1358-x PMC6693180

[11] Mei Q, Ye LJ, Lin H, et al. CUL4A promotes the invasion of cervical cancer cells by regulating NF-κB signaling pathway. Eur Rev Med Pharmacol Sci. 2020 Oct;24(20):10403–10409. 33155196 10.26355/eurrev_202010_23390

[12] Ni W, Zhang Y, Zhan Z, et al. A novel lncRNA uc.134 represses hepatocellular carcinoma progression by inhibiting CUL4A-mediated ubiquitination of LATS1. J Hematol Oncol. 2017 Apr 19 10(1):91. 28420424 10.1186/s13045-017-0449-4 PMC5395742

[13] Hung MS, Chen YC, Lin P, et al. Cul4A Modulates Invasion and Metastasis of Lung Cancer Through Regulation of ANXA10. Cancers (Basel). 2019 May 2 11(5):618. 10.3390/cancers11050618 PMC6562482 31052599

[14] Yi LJ, Yi LJ, Ding N, et al. CUL4A promotes proliferation and inhibits apoptosis of colon cancer cells via regulating Hippo pathway. Eur Rev Med Pharmacol Sci. 2020 Oct;24(20):10518–10525. 33155207 10.26355/eurrev_202010_23404

[15] Chen HH, Fan P, Chang SW, et al. NRIP/DCAF6 stabilizes the androgen receptor protein by displacing DDB2 from the CUL4A-DDB1 E3 ligase complex in prostate cancer. Oncotarget. 2017 Mar 28 8(13):21501–21515. 28212551 10.18632/oncotarget.15308 PMC5400601

[16] Jin X, Ma YC, Zhu WY, et al. CUL4A expression is associated with tumor stage and prognosis in nasopharyngeal carcinoma. Medicine (Baltimore). 2019 Dec;98(51):e18036. 31860954 10.1097/MD.0000000000018036 PMC6940123

[17] Li M, Huang H, Cheng F, et al. miR-141-3p promotes proliferation and metastasis of nasopharyngeal carcinoma by targeting NME1. Adv Med Sci. 2020 Sep;65(2):252–258. 32299022 10.1016/j.advms.2020.03.005

[18] Chan KC, Ting CM, Chan PS, et al. A novel Hsp90 inhibitor AT13387 induces senescence in EBV-positive nasopharyngeal carcinoma cells and suppresses tumor formation. Mol Cancer. 2013 Oct 24 12(1):128. 10.1186/1476-4598-12-128 PMC3834878 24156782

[19] Yu X, Lin XJ, Wang S, et al. Antitumor Efficacy of Huqizhengxiao (HQZX) Decoction Based on Inhibition of Telomerase Activity in Nude Mice of Hepatocarcinoma Xenograft. Integr Cancer Ther. 2018 Dec;17(4):1216–1224. 29978739 10.1177/1534735418785999 PMC6247564

[20] Gallo-Oller G, Ordoñez R, Dotor J. A new background subtraction method for Western blot densitometry band quantification through image analysis software. J Immunol Methods. 2018 Jun;457:1–5. 29522776 10.1016/j.jim.2018.03.004

[21] Livak KJ, Schmittgen TD. Analysis of relative gene expression data using real-time quantitative PCR and the 2(-Delta Delta C(T)) Method. Methods (San Diego. Calif). 2001 Dec;25(4):402–408. 10.1006/meth.2001.1262 11846609

[22] Pan S, Deng Y, Fu J, et al. TRIM52 promotes colorectal cancer cell proliferation through the STAT3 signaling. Cancer Cell Int. 2019;19(1):57. 30918473 10.1186/s12935-019-0775-4 PMC6419475

[23] Sun S, Gao T, Pang B, et al. RNA binding protein NKAP protects glioblastoma cells from ferroptosis by promoting SLC7A11 mRNA splicing in an m(6)A-dependent manner. Cell Death Dis. [2022 Jan 21];13(1):73. 35064112 10.1038/s41419-022-04524-2 PMC8783023

[24] He L, Zhu C, Jia J, et al. ADSC-Exos containing MALAT1 promotes wound healing by targeting miR-124 through activating Wnt/β-catenin pathway. Biosci Rep. 2020 May 29;40(5). 10.1042/BSR20192549. PMC7214401 32342982

[25] Wu D, Kang L, Tian J, et al. Exosomes Derived from Bone Mesenchymal Stem Cells with the Stimulation of Fe(3)O(4) Nanoparticles and Static Magnetic Field Enhance Wound Healing Through Upregulated miR-21-5p. Int J Nanomedicine. 2020;15:7979–7993. 33116513 10.2147/IJN.S275650 PMC7585514

[26] Wang C, Cheng Y, Liu H, et al. Pectolinarigenin Suppresses the Tumor Growth in Nasopharyngeal Carcinoma. Cell Physiol Biochem. 2016;39(5):1795–1803. 27744436 10.1159/000447879

[27] Franz M, Rodriguez H, Lopes C, et al. GeneMANIA update 2018. Nucleic Acids Res. 2018 Jul 2 46(W1):W60–w64. 29912392 10.1093/nar/gky311 PMC6030815

[28] Guo R, Mao YP, Tang LL, et al. The evolution of nasopharyngeal carcinoma staging. Br J Radiol. 2019 Oct;92(1102):20190244. 31298937 10.1259/bjr.20190244 PMC6774596

[29] Chen YP, Chan ATC, Le QT, et al. Nasopharyngeal carcinoma. Lancet (London. England). [2019 Jul 6];394(10192):64–80. 10.1016/S0140-6736(19)30956-0 31178151

[30] Song Y, Xiao H, Yang Z, et al. The predictive value of pre- and post-induction chemotherapy plasma EBV DNA level and tumor volume for the radiosensitivity of locally advanced nasopharyngeal carcinoma. EXCLI J. 2017;16:1268–1275. 29333129 10.17179/excli2017-752 PMC5763095

[31] Ji Y, Li H, Wang F, et al. PPARβ/δ Agonist GW501516 Inhibits Tumorigenicity of Undifferentiated Nasopharyngeal Carcinoma in C666-1 Cells by Promoting Apoptosis. Front Pharmacol. 2018;9:648. 30002625 10.3389/fphar.2018.00648 PMC6031703

[32] Wakae K, Kondo S, Pham HT, et al. EBV-LMP1 induces APOBEC3s and mitochondrial DNA hypermutation in nasopharyngeal cancer. Cancer Med. 2020 Oct;9(20):7663–7671. 32815637 10.1002/cam4.3357 PMC7571841

[33] Tsao SW, Tsang CM, Lo KW. Epstein-Barr virus infection and nasopharyngeal carcinoma. Philos Trans R Soc London, Ser B. 2017 Oct 19;372:20160270. 28893937 10.1098/rstb.2016.0270 PMC5597737

[34] Schnappauf O, Aksentijevich I. Mendelian diseases of dysregulated canonical NF-κB signaling: from immunodeficiency to inflammation. J Leukoc Biol. 2020 Aug;108(2):573–589. 32678922 10.1002/JLB.2MR0520-166R

[35] Liang Y, Feng G, Wu L, et al. Caffeic acid phenethyl ester suppressed growth and metastasis of nasopharyngeal carcinoma cells by inactivating the NF-κB pathway. Drug Des Devel Ther. 2019;13:1335–1345. 10.2147/DDDT.S199182 PMC6499142 31118570

[36] Yang D, Liang T, Gu Y, et al. Protein N-arginine methyltransferase 5 promotes the tumor progression and radioresistance of nasopharyngeal carcinoma. Oncol Rep. 2016 Mar;35(3):1703–1710. 26708443 10.3892/or.2015.4513

[37] Sloan SL, Renaldo KA, Long M, et al. Validation of protein arginine methyltransferase 5 (PRMT5) as a candidate therapeutic target in the spontaneous canine model of non-Hodgkin lymphoma. PloS one. 2021;16(5):e0250839. 33989303 10.1371/journal.pone.0250839 PMC8121334

[38] Yuan Y, Nie H. Protein arginine methyltransferase 5: a potential cancer therapeutic target. Cell Oncol (Dordr). 2021 Feb;44(1):33–44. 33469838 10.1007/s13402-020-00577-7 PMC12980669

